# Lipidomics Identified Lyso-Phosphatidylcholine and Phosphatidylethanolamine as Potential Biomarkers for Diagnosis of Laryngeal Cancer

**DOI:** 10.3389/fonc.2021.646779

**Published:** 2021-06-28

**Authors:** Bo Yu, Jizhe Wang

**Affiliations:** Department of Otolaryngology Head and Neck Surgery, The Second Hospital of Dalian Medical University, Dalian, China

**Keywords:** laryngeal cancer, lipidomics, UHPLC-mass spectrometry, biomarker, lipid metabolic abnormality

## Abstract

**Background:**

Laryngeal cancer (LaC) remains one of the most common tumors of the respiratory tract with higher incidence in men than in women. The larynx is a small but vital organ on the neck. The dysfunction of the larynx can cause serious health problems such as hoarseness, respiratory distress, and dysphonia. Many lipids (e.g. phospholipid, cholesterol, fatty acid) have been recognized as a crucial role in tumorigenesis. However, the lipid biomarkers are lacking and the lipid molecular pathogenesis of LaC is still unclear.

**Methods:**

This study aims to identify new LaC-related lipid biomarkers used for the diagnosis or early diagnosis of LaC and to uncover their molecular characteristics. Thus, we conducted serum and tissue nontargeted lipidomics study from LaC patients (n = 29) and normal controls (NC) (n = 36) *via* ultra-high performance liquid chromatography (UHPLC) coupled with high resolution mass spectrometry (HRMS). Multivariate and univariate statistics analyses were used to discriminate LaC patients from NC.

**Results:**

As expected, a lipid panel including LPC (16:0) and PE (18:0p_20:4) was defined to distinguish the LaC patients from healthy individuals with very high diagnosis performance (area under the curve (AUC) value = 1.000, sensitivity value = 1.000, and specificity value = 1.000). In addition, the levels of Cer, CerG1, SM, PC, PC-O, PE, PI, PS, and ChE in the LaC group significantly increased as compared with the NC group. However, the levels of LPC, LPC-O, LPE, LPE-p, and DG in the LaC group significantly deceased when the one was compared with the NC group. Among significantly changed lipid species, lysophospholipids containing a palmitoyl chain or an arachidonic acid acyl chain remarkably decreased and phospholipids including a palmitoyl chain or an arachidonic acid acyl chain increased in the LaC patients.

**Conclusion:**

Our results not only indicate that lipidomics is powerful tool to explore abnormal lipid metabolism for the laC, but suggest that lysophospholipids and phospholipids may serve as potential biomarkers for diagnosis of LaC.

## Introduction

Laryngeal cancer is the most common head and neck cancer, causing heavy health care and economic burdens. Currently, the Global Burden of Disease Cancer Collaboration estimated the prevalence of LaC to be 21.1%, with a male to female ratio of 5:1, and approximately 10% of patients in metastatic or end-stage (1). Notably, over the past 30 years, the burden of this malignancy (expressed in years lived with disability) has increased by nearly 25% (2).

Some LaC patients have been diagnosed at the advanced stage and have an unsatisfactory treatment effect (3). Early detection of LaC is essential for treating this disease. Although some imaging methods, for example computed tomography (CT) (4), positron emission tomography (PET) scan (5), and magnetic resonance imaging (MRI) (6), are commonly used in the screening and detection of LaC, current imaging methods are challenged by problems related to availability of primary healthcare workers capable of assessing images. Thus, there is still an urgent need to identify novel biomarkers for LaC screening or detection.

Lipidomics, focusing on comprehensive profiling of lipids in complex biological matrice, is a powerful tool to identify disease-related lipid biomarkers contributing to diagnosis of disease, and to explore disordered lipid metabolism in the development of diseases. It has been widely applied in many studies, such as diabetes (7, 8), lung cancer (9), liver cancer (10, 11), oral cancer (12) and so on. Serum biomarkers related to head and neck cancer was explored by Yonezawa et al. (13), and they revealed that the levels of several metabolites associated with glycolytic pathways significantly increased in patients with head and neck cancer. However, the levels of several amino acids (e.g., serine, methionine, valine, and thyroxine) were too low. Plasma lipidomics was performed and revealed tamoxifen-induced alteration of the hepatic lipid profile and its association with the lipid profile (14). AA-containing PCs might have potential utility as novel and predictive biomarkers for tamoxifen-induced hepatic steatosis and phospholipidosis. Unfortunately, no laryngeal cancer-associate lipidomic study has yet been performed.

**Figure 1:**
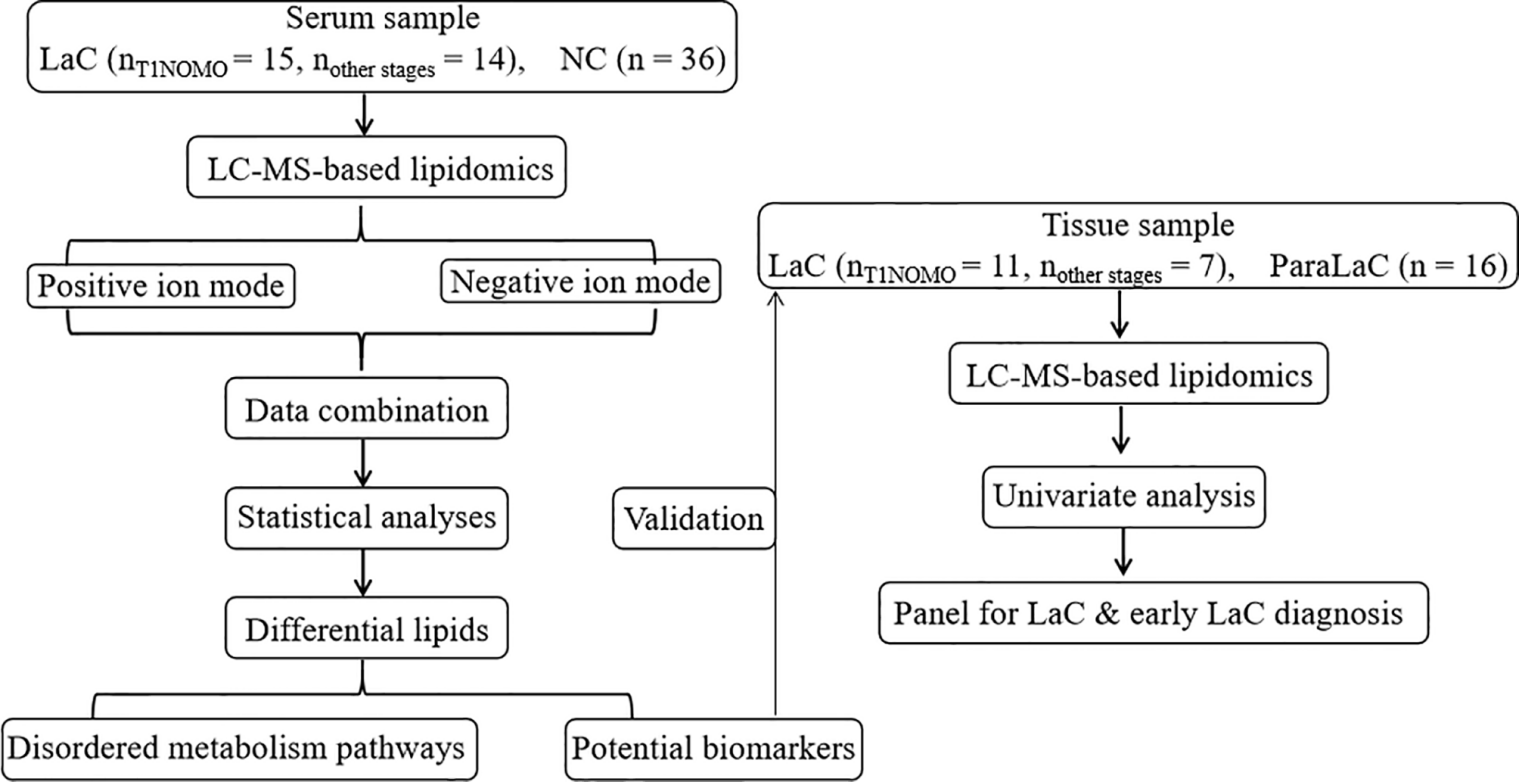
Flowchart of the study design.

The aims of this study are to identify reliable serum biomarkers in diagnosing LaC and early-stage LaC, and to comprehensively elucidate the abnormal lipid metabolism related to the onset and development of LaC utilizing nontargeted lipidomics method based on UHPLC/Q-TOF mass spectrometry. Thus, a total of 65 participants were enrolled to discover a novel lipid panel and test its diagnosis performance, and to explore abnormal lipid metabolism pathways related to LaC (**Figure 1**).

## Materials and Methods

### Clinical Samples

Serum samples collected from 29 patients diagnosed with laryngeal cancer (LaC) and a set of 36 sex-age matched normal controls (NC) were from the Second Hospital of Dalian Medical University (Dalian, China) during the period 2018 to 2020. These subjects were in the age range of 53–80 years. Among these LaC serum samples, 15 cases were in the early stage of laryngeal cancer, i.e. T1NOMO stage. The LaC serum samples were collected before surgical resection and then stored at −80°C until lipidomics analysis. Eighteen LaC tissue (LaCT) and 16 adjacent noncancerous tissue (ANT) samples were also provided by the Second Hospital of Dalian Medical University (Dalian, China). Among these LaC tissue samples; 11 cases were in T1NOMO stage. This research was approved by the Second Hospital of Dalian Medical University Institutional Ethics Review Board, and all participants provided written informed consent.

### Total Lipid Extraction

Serum samples taken out of the −80° refrigerator were thawed and homogenized by vortex. A total of 300 µl of cold methanol (HPLC-grade, Merck, Darmstadt, Germany) including seven lipid internal standards (i.e., FFA (16-d3), LPC (19:0), Cer (d18:1/17:0), SM (d18:1/12:0), PC (19:0/19:0), PE (17:0/17:0), TG (15:0/15:0/15:0)), purchased from Avanti Polar Lipids (Alabaster, USA), was added into 40 µl of each serum sample, followed by a 30-s vortex. Then, 1 ml of HPLC-grade tert-butyl methyl ether (MTBE) was added and the mixture was homogenized for 10 min. After that, 300 µl of ultrapure water (Milli-Q system, Millipore, Billerica, MA) was added. Next, the mixture was vortexed and centrifuged (4°C, 14,000*g*) until to form the two-phase system. About 400 µl aliquot of the upper layer were drawn and dried in a vacuum centrifuge and then stored at −80°C prior to LC-MS analysis. Quality control (QC) samples were made by mixing equal aliquots from each serum sample and pretreated using the same procedures as that description of the real samples. The QC sample was inserted into the batch after every six real samples to assess the reproducibility of the preparation and the LC–MS system.

Tissue sample were weighed. About 400 µl of cold methanol including seven lipid internal standards was added, followed by the homogenization at 25 Hz for 2 min on a mixer mill MM400 (Retsch, Haan, Germany). And then 1 ml of MTBE was added and the mixture was vibrated for 15 min. After that, 300 µl of ultrapure water was added. The mixture was vortexed and centrifuged (4°C, 14,000*g*) until to form the two-phase system. About 200 µl aliquot of the upper layer were drawn and dried in a vacuum centrifuge and then stored at −80 °C before LC–MS profiling. Details on lipid internal standards that were added to samples before lipidome extraction are summarized in **Table S1**.

### Lipidomics Analysis

The lipidome was analyzed using Waters ACQUITY UPLC (Waters, Milford, USA) coupled with a Triple TOF 5600 Plus mass spectrometer (AB SCIEX, USA) system. Before LC–MS analysis, the lyophilized samples were reconstituted in the mixed solution including CH_2_Cl_2_ and MeOH (2:1 v/v) and then diluted in the ACN-MeOH-H_2_O solution (65:30:5 v/v/v). Next, 5 µl of the diluted sample was separated on the C8 ACQUITYTM column (100 × 2.1 mm, 1.7 µm), (Waters, Milford, MA, USA). The column temperature was kept 60°C. The elution rate was set 0.3 ml/min. The mobile phase A was ACN:H2O (6:4 v/v) and the mobile phase B, was IPA: ACN (9:1 v/v), both containing 10 mM ammonium acetate. The initial elution gradient began with 50% B, kept for 1.5 min, followed by a linear increase to 85% B at 9.0 min, then reached at 100% B within 0.1 min, and maintained for 1.9 min. Lastly, it returned to 50% B within 0.1 min and held for 1.9 min to equilibrate column. The scanned m/z range of MS signal was 200–1,250 Dalton in both positive and negative ion modes. The capillary voltages of the positive and negative ion modes were set at 5.5 and −4.5 kV respectively. The interface heater temperature was set at 500 and 550°C for positive and negative ion modes, respectively.

### Identification of Lipids

The structural compositions (e.g. PC18:0_20:4) of lipids were identified when characteristic ions and fatty acyl fragments appeared in the MS/MS spectrum. For those without fatty acyl fragments but with only characteristic ionic information, they would be annotated with total no. of carbon atoms and double bonds of acyl chains, e.g., PC36:0. Furthermore, for lipids that could not produce MS/MS fragments, extraction ion chromatogram (EIC) was performed based on an in-house lipid database for these lipids with a mass tolerance of 10 ppm to obtain the observed m/z and *t*_R_. And then, these lipid candidates were further confirmed by comparing the relative *t*_R_ between the known lipids and the candidate peaks within a given lipid class.

### Data Processing and Statistical Analysis

The lipids were identified according to previous published paper (15). The detected lipids were quantified *via* MultiQuant™ 2.1 (AB SCIEX, Concord, Canada) software with a mass error of ±0.05 Da and *t*_R_ shift of ±0.25 min. Lipidomics data were normalized by the corresponding internal standards.

The supervised partial least-squares discriminant analysis (PLS-DA) was performed on SIMCA-P software (13.0 version, Umetrics Umeå, Sweden), in Pareto scaling mode, which suppresses noise interference *via* dividing each variable by the square root of the standard deviation. Nonparametric test in Wilcoxon, Mann–Whitney test mode, was implemented to identify significantly altered lipids (*p <*0.01 & FDR <0.05) by the comparisons between the LaC and NC groups and heatmap was produced by the open-source software MultiExperiment Viewer (MeV, version 4.9.0). Receiver operating characteristic (ROC) curves of a binary logistic regression using SPSS software version 19 (SPSS, Inc.) were performed. The bar graph of the significantly differential lipid (sub)classes was drawn on the GraphPad Prism software (6.0 version).

## Results

### Clinical Characteristics of the Laryngeal Cancer Patients and the Normal Controls

The detailed clinical information of the LaC and NC groups are provided in **Table 1**. Laryngeal cancer staging was performed in 29 patients who underwent laryngeal cancer resection according to the 8th edition of the AJCC Cancer Staging Manual (16). In this study, T1NOMO stage of laryngeal was also considered for diagnosing the disease at an early stage. The age and sex between the LaC group and the NC group are matched as much as possible.

**Table 1:** Characteristics of the subjects for lipidomics analyses.

Characteristics	Serum sample		Tissue sample	
	LaC	NC	LaC	ParaLaC
No.	29	36	18	16
Sex (male/female)	29/0	36/0	18/0	16/0
Age (years)	61.56 ± 8.96	56.22 ± 16.07^–^ ^a^	64.59 ± 9.58	62.08 ± 8.05
BMI (kg/m^2^)	24.01 ± 3.71		24.30 ± 3.06	24.84 ± 2.74
FPG (mmol/L)	5.64 ± 0.87	5.71 ± 0.91	5.78 ± 0.97	5.70 ± 1.09
TB (µmol/L)	13.65 ± 4.64	16.21 ± 7.10	13.63 ± 4.12	13.87 ± 6.51
HCT (%)	43.48 ± 3.48	43.92 ± 4.15	43.85 ± 4.05	44.48 ± 2.59
HGB (g/L)	146.36 ± 13.22	148.54 ± 13.85	147.47 ± 16.06	150.50 ± 10.52
MCHC (g/L)	336.44 ± 10.23	338.11 ± 6.58	335.82 ± 10.39	338.17 ± 11.03
MCH (pg)	31.14 ± 2.43	31.64 ± 1.27	31.64 ± 1.82	31.84 ± 1.61
MCV (fL)	92.54 ± 6.27	93.48 ± 3.91	94.22 ± 4.86	94.15 ± 3.87
PLT (10^9^/L)	223.92 ± 56.60	215.23 ± 65.20	213.29 ± 43.17	220.92 ± 34.78
WBC (10^9^/L)	6.66 ± 1.66	7.33 ± 1.88	6.56 ± 1.28	6.77 ± 1.26
RBC (10^12^/L)	4.72 ± 0.50	4.69 ± 0.41	4.60 ± 0.55	4.71 ± 0.33
Urea (mmol/L)	6.09 ± 1.74	5.86 ± 2.09	6.89 ± 2.01	6.51 ± 1.78
UricAcid (µmol/L)	381.20 ± 82.41	360.34 ± 83.75	391.88 ± 84.75	405.41 ± 99.99

a NC group lacks of BMI information. Data represent mean ± SD.

### Lipidome Fingerprinting Between Patients With LaC and the NC

Lipidomics profiling was performed for comparative analyses of the serum samples collected from the LaC patients (n = 29) and the NC subjects (n = 36). The total ion current chromatograms of serum were shown in (**Figure S1**) for LaC and NC subjects in both positive and negative ion modes, respectively. In this study, 390 lipids were identified by exact mass-to-charge ratio (M/Z), retention time (*t*_R_), and/or characteristic fragments. And the list of exact m/z values and retention times and characteristic fragments of all the lipid species identified was provided in **Table S2**. Among these identified lipids, 17 common lipid (sub)classes, mainly containing FA, LPC, LPE, PC, PE, Cer, SM, DG, and TG, were identified (**Figures S2A, B**). The QC sample was inserted into the analytical batch after six real samples to monitor the lipidomics data quality. In **Figure S2C**, relative standard deviations (RSD) of 47 and 87% of the detected lipids in all serum QC samples were less than 10 and 20%, respectively. The percentage of the identified lipids with RSD below 30% reached at 98%, which confirmed the analytical stability of the LC-MS-based lipidomics method used to acquire the lipidome data. And the detected lipids with RSD less than 30% were used for the follow-up statistical analysis.

**Figure 2:**
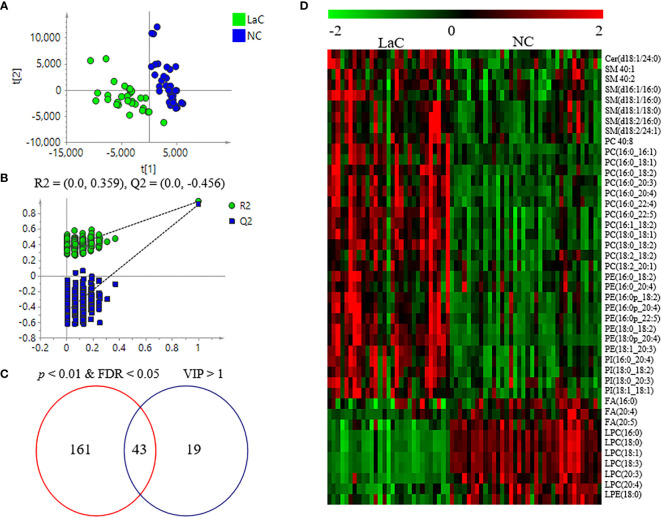
**(A)** Score plots of PLS-DA between LaC and NC for lipidomics data from serum samples. **(B)** Cross validation of PLS-DA model between LaC and NC for lipidomics analyses. **(C)** Venn diagram shows the differential lipids between the LaC group and the NC group in serum samples. **(D)** Heatmap overview of the 43 differential lipids in distinguishing patients with LaC from the normal controls.

A supervised PLS-DA model was made based on those identified lipids from serum samples to explore whether abnormalities in lipid metabolism occurred during the development of laryngeal cancer. In **Figure 2A**, we could find that the LaC group was apparently distinguished from the NC group. Subsequently, 200 times of permutations were operated to evaluate whether the PLS-DA model is over-fitting. In **Figure 2B**, R^2^ = (0.0, 0.359) and Q^2^ = (0.0, −0.456) shown that this model is stable. These results implied that substantial lipidome alternations occurred underlying the onset and development of LaC. Sixty-two lipids with variable importance for the projection (VIP) >1.0 were recognized as key variables that contribute to the classifications.

### Defining of Potential Lipid Biomarkers for LaC

At present, the identification of novel serum markers for diagnosing LaC remains a vital task, especially for the early detection of LaC (17). In the present study, we employed the nontargeted lipidomics method, identifying as many lipids as possible, to screen biomarkers.

**Figure 3:**
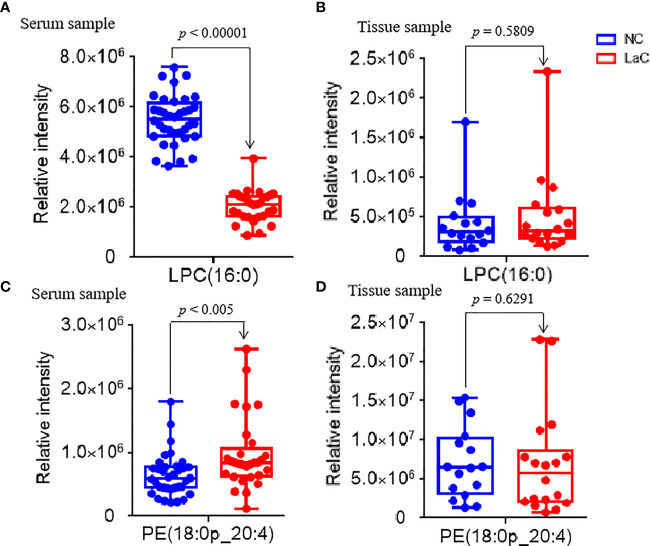
**(A, C)** Serum relative intensity of defined potential biomarkers of LPC(16:0) and PE(18:0p_20:4), respectively. **(B, D)** Tissue relative intensity of defined potential biomarkers of LPC(16:0) and PE(18:0p_20:4), respectively.

To explore significantly differential lipid species between the LaC group and the NC group, a univariate analysis (non-parameter test) was performed based on the lipidomics data from the LaC and NC groups. The levels of 204 lipids were noted significant changes between the LaC patients and the NCs (*p* value <0.01 and false discovery rate (FDR) value <0.05). The information of the differential lipids are provided in **Table S3**. Finally, 43 of these lipids exhibited *p <*0.01, FDR <0.05 and VIP >1.0 in the two comparisons (**Figure 2C**). In addition, heatmap visualization based on 43 differential lipids was performed to obtain an overview of the pattern of lipidomic alterations with the LaC development in a clinical setting (**Figure 2D**). Subsequently, for these 43 potential lipid biomarkers, the best model was constructed by a binary logistic regression analysis with an optimized algorithm of the forward stepwise (Wald). At last, the combination of LPC (16:0) (**Figures 3A, B**) and PE (18:0p_20:4) (**Figures 3C, D**) was defined as the ideal lipid panel in distinguishing patients with LaC from normal controls.

**Figure 4:**
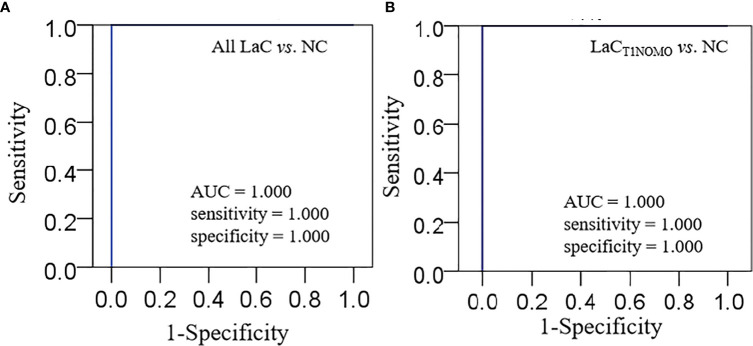
**(A)** Characterization of ROC curve of lipid panel in the serum samples from the LaC group and NC group. **(B)** Characterization of ROC curve of lipid panel in the serum samples from the LaC_T1NOMO_ group and NC group. AUC, Area under curve; Lipid panel, LPC(16:0) and PE(18:0p_20:4).

This lipid panel had high diagnostic performances, such as AUC value = 1.000, sensitivity value = 1.000, and specificity value = 1.000 in the discrimination of LaC from NC in the serum sample, respectively (**Figure 4A**). Furthermore, the serum lipid panel had a perfect performance in identifying the LaC_T1NOMO_ at early-stage LaC from the NC group, such as AUC, sensitivity, and specificity values of 1.000, 1.000, and 1.000, respectively (**Figure 4B**). Collectively, the serum lipid panel separated LaC from the NC with very high performance. Moreover, this lipid panel effectively discriminated patients with LaC_T1NOMO_ from the NC, highlighting the early diagnostic potential of this lipid biomarker pane.

### Characteristics of Lipid (Sub)Classes Between the LaC and NC Groups

Lipid analysis between the LaC and NC groups was further investigated at the level of a given lipid (sub)class. For this purpose, the total content of all lipid species within a given (sub)class was compared between the LaC and NC groups according to Student T- test (*p <*0.05). The results shown that the relative contents of Cer, CerG1, SM, PC, PC-O, PE, PI, PS, and ChE in the LaC group significantly accumulated relative to the NC group. The levels of LPC, LPC-O, LPE, LPE-p, and DG in the LaC group significantly deceased when the one was compared with the NC group (**Table 2**).

**Table 2:** Fold changes and *p* values of each lipid class between patients with LaC and controls.

Lipid class	Serum sample (LaC vs NC)		Tissue sample (LaCT vs ANT)	
	Fold change	*p *value	Fold change	*p *value
FA	0.95	5.0E−01	1.33	4.9E−01
OAHFA	1.04	8.0E−01	1.50	5.7E−01
Cer	1.15	3.2E−02	1.27	2.6E−01
CerG1	1.28	5.3E−04	1.16	5.4E−01
CerG2	1.06	4.7E−01	0.88	7.3E−01
SM	1.35	2.1E−06	1.17	2.5E−01
LPC	0.42	1.2E−22	1.37	3.1E−01
LPC-O	0.29	3.3E−26	1.65	1.3E−01
LPE	0.84	2.1E−02	1.09	7.5E−01
LPE-p	0.26	5.0E−15	1.48	3.5E−01
PC	1.84	2.9E−11	1.15	3.2E−01
PC-O	1.53	1.1E−06	1.12	5.0E−01
PE	1.69	1.1E−04	1.06	8.2E−01
PE-p	1.30	7.9E−02	1.11	6.3E−01
PI	1.66	5.2E−06	5.72	1.1E−01
PS	1.35	1.2E−06	3.25	1.6E−01
DG	0.47	4.4E−08	2.00	2.0E−01
TG	1.09	5.6E−01	0.27	1.4E−01
ChE	1.18	7.0E−03	1.51	2.3E−01

**Figure 5:**
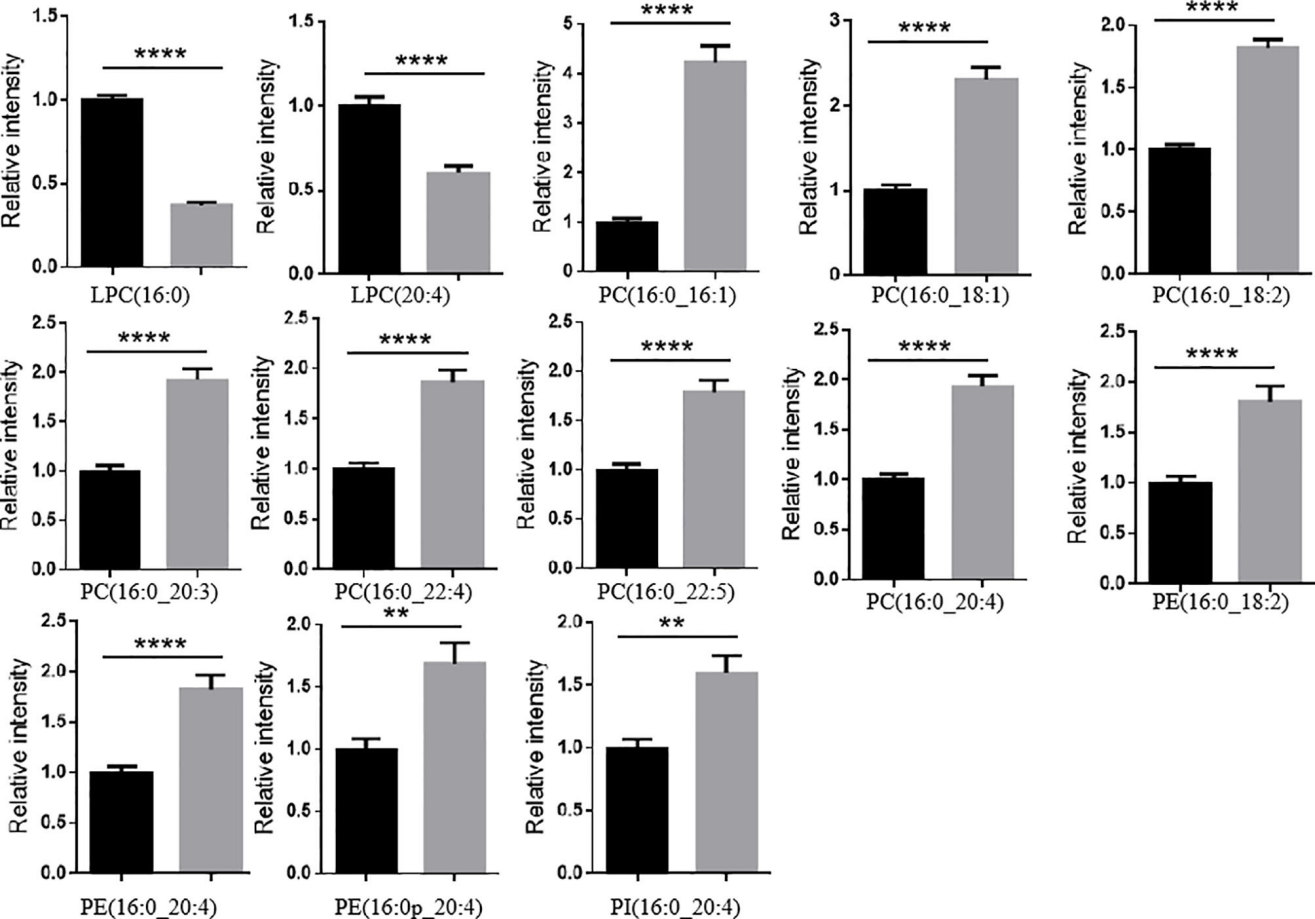
LPLs containing a palmitoyl chain or an arachidonic acid acyl chain significantly decreased and PLs containing a palmitoyl chain or an arachidonic acid acyl chain mostly significantly increased in LaC vs. NC in serum samples. ****p < 0.00001, **p < 0.001.

Another finding of interest was that the levels of large amount of PLs (e.g., PC, PE, and PI) with an arachidonic acid acyl chain and/or a palmitoyl chain significantly increased in LaC vs NC, and the levels of LPCs with an arachidonic acid acyl chain and/or a palmitoyl chain significantly decreased in LaC vs NC (**Figure 5**).

## Discussion

In this study, there were no significant differences in most clinical characteristics (**Table 1**) between the LaC and NC groups. Clinical outcomes clearly do not explain the observable phenotypic differences. Lipids are vital in cellular functions due to they are the essential components of the membrane structure, key regulators in signal transduction and energy storage (18). Abnormal lipid metabolism is growingly recognized as a hallmark of tumors and associated with the onset and development of many human diseases (19). Therefore, we performed a comprehensive lipidome analysis of larynx tumor between patients with LaC and the normal controls. As far as we know, a systematic evaluation of tumor lipid metabolism in LaC patients was reported for the first time.

The relative levels of most Cer and SM lipids significantly increased in the LaC patients when they were compared with the healthy controls. Ceramide is bioactive lipids of the sphingolipid pathway and play essential roles in cell signaling. Ceramide has been shown to be involved in stress-related cellular responses and apoptosis (20, 21). The imbalance of ceramide metabolism will greatly influence the physical and chemical properties of cells, leading to cellular dysfunctions. Many studies reported that ceramide metabolism altered in numerous cancers characterized by an increase of the Cer profile in cancer cells and tumor tissue (10, 22, 23). We speculated that the significant elevation in the level of the Cers in LaC patients could have resulted from the upregulated expression of the enzymes related to the synthesis of ceramide. It was reported that ceramide synthase in a salvage pathway was highly activated in several different tumors, such as human colon cancer (24), human non-small-cell lung cancer (25). *In vivo*, Cer can be also generated by the hydrolysis of SM through the actions of sphingomyelinases. Modulation of endogenous Cer levels is considered as a new therapeutic target for anti-cancer intervention strategies (26). In all, we hypotheses that reducing Cer biosynthesis or preventing from converting SM to Cer could inhibit LaC progression.

It is well known that PL is one of the most important components of a mammalian membrane bilayer. PC is the mostly predominant component of PLs for biological membrane. Some reports have shown that PC metabolism is altered in the onset and development of many cancers, characterized by an elevation of PC (27–29). We deduced that the significant increase in PC may be due to an imbalance between PC and PE. Increased PC and an imbalance between PC and PE have been reported to be associated with obesity and NAFLD (30, 31), both of which are also associated with LaC occurrence. In addition, PC and LPC mutually convert, upregulating PC level which may come from LPC conversion. This point can be supported by the significant decrease in the level of LPC for LaC patients. Altogether, disordered PC lipid metabolism is closely associated with the development of Lac.

In this study, we also found that PLs with PUFA, in particular arachidonic acid residues, significantly increased in LaC patients. Arachidonic acid is one of major PUFAs in mammals. Long-chain acyl-coenzyme A synthetase 4 (ACSL4), shows preferential use of AA as its substrate and plays a role in the remodeling of AA-containing glycerolphospholipids by binding free AA. In consideration of significant increase of AA-residue-enriched PLs (e.g., PC(16:0_20:4), PE(16:0_20:4), PE(16:0p_20:4), PE(18:0p_20:4), PI(16:0_20:4), etc.), and the level of AA, so-called FA (20:4) significantly decreased in LaC serum, we speculated that ACSL4 may be activated and thereby prompt PLs accumulation, which associated with a greater degree of carcinogenesis in LaC tumor cells characterized by very abundant mitochondria. Of course, further investigation should be performed to explore our findings.

In summary, using nontargeted lipidomics method based on UHPLC-HRMS, we successfully identified a lipid panel [including LPC(16:0) and PE(18:0p_20:4)] that can effectively diagnose LaC from their cohort of healthy controls. Similar, this lipid panel shows ultrahigh performance in detection of the early-stage LaC from the healthy volunteers. To our best knowledge, this study provides the first evidence of a systematic alteration in lipid composition between LaC and NC groups. Cer, SM, and AA-enriched PLs showing close association with LaC, may be potential biomarkers and become potential targets for LaC. Out of consideration for the given small sample size, and to ensure the plausibility of our study results, further studies based on large-scale clinical samples and on the expression of related lipid enzymes will be required.

## Data Availability Statement

The datasets presented in this study can be found in online repositories. The names of the repository/repositories and accession number(s) can be found below: https://www.ebi.ac.uk/metabolights/MTBLS2683.

## Ethics Statement

The studies involving human participants were reviewed and approved by the Second Hospital of Dalian Medical University. The patients/participants provided their written informed consent to participate in this study.

## Author Contributions

JW and BY conceived and designed the project. BY collected the clinical samples, performed the experiments, and analyzed the data. BY and JW wrote and improved the manuscript. JW and BY are responsible for the integrity and accuracy of the data in the research. All authors contributed to the article and approved the submitted version.

## Funding

This study was supported by the National Natural Science Foundation of China Grants (No. 11772087), and the Joint Fund Project of Collaborative Innovation Center for Individualized Diagnosis and Treatment (dy2yhws201805).

## Conflict of Interest

The authors declare that the research was conducted in the absence of any commercial or financial relationships that could be construed as a potential conflict of interest.

## Abbreviations

LaC, laryngeal cancer; NC, normal control; MTBE, tert-butyl methyl ether; ACN, d acetonitrile; MeOH, methanol; IPA, isopropanol; PL, glycerophospholipid; AA, arachidonic acid; UHPLC, ultra-high performance liquid chromatography; HRMS, high resolution mass spectrometry; QC, quality control; RSD, relative standard deviation; FA, fatty acid; OAHFA, (O-acyl)-1-hydroxy fatty acid; Cer, ceramide; CerG1, glucosylceramide; CerG2, galactosylceramide; SM, sphingomyelin; LPC, lyso-glycerophosphatidylcholine; LPC-O, LPC with alkyl substituents; PC, glycerophosphatidylcholine; PC-O, PC with alkyl and alkenyl substituents; LPE, lyso-glycerophosphatidylethanolamine; LPE-p, LPE with alkenyl substituents; PE, glycerophosphatidylethanolamine; PE-p, PE with alkenyl substituents; PI, glycerophosphatidylinositol; PG, glycerophosphatidylglycerol; PS, glycerophosphatidylserine; SL, sphingolipid; ChE, cholesterol ester; DG, diacylglycerol; TG, triacylglycerol; PLS-DA, partial least squares-discrimination analysis; LysoPL, lyso-glycerophospholipid; PUFA, polyunsaturated FA.
