# Supplementary material for: Lipidomics Identified Lyso-Phosphatidylcholine and Phosphatidylethanolamine as Potential Biomarkers for Diagnosis of Laryngeal Cancer
Source: Front Oncol. 2021 Jun 28;11:646779. doi: 10.3389/fonc.2021.646779 (PMC8273650; doi:10.3389/fonc.2021.646779)
Supplement: Supplementary file 1 [file DataSheet_1.doc]

Lipidomics Identified Lyso-Phosphatidylcholine and Phosphatidylethanolamine as Potential Biomarkers for Diagnosis of Laryngeal Cancer

Bo Yu ^1^, Jizhe Wang ^1*^

^1^ Department of Otolaryngology Head and Neck Surgery, The Second Hospital of Dalian Medical University, Dalian, Liaoning 116023, P.R. China.

^*^ Corresponding author at:

Jizhe Wang. M.D.

Department of Otolaryngology Head and Neck Surgery

The Second Hospital of Dalian Medical University

Dalian. 116023. China.

E-mail addresses: 270573617@qq.com

Contents

Fig. S1 The total ion current chromatograms of serum and tissue from LaC and NC subjects in both positive and negative ion modes........................................................S3

Fig. S2 Distribution of all identified lipid species (subclass, no. of lipids) and data evaluation.....................................................................................................................S4

Table S1 Information on lipid internal standards that were spiked in sample matrix prior to lipidom extraction...........................................................................................S5

Table S2. The list of exact m/z values and retention times of all the lipid species identified......................................................................................................................S5

Table S3 The details of the differential lipids contributed to distinguishing LaC from NC.........................................................................................................................S6-S10


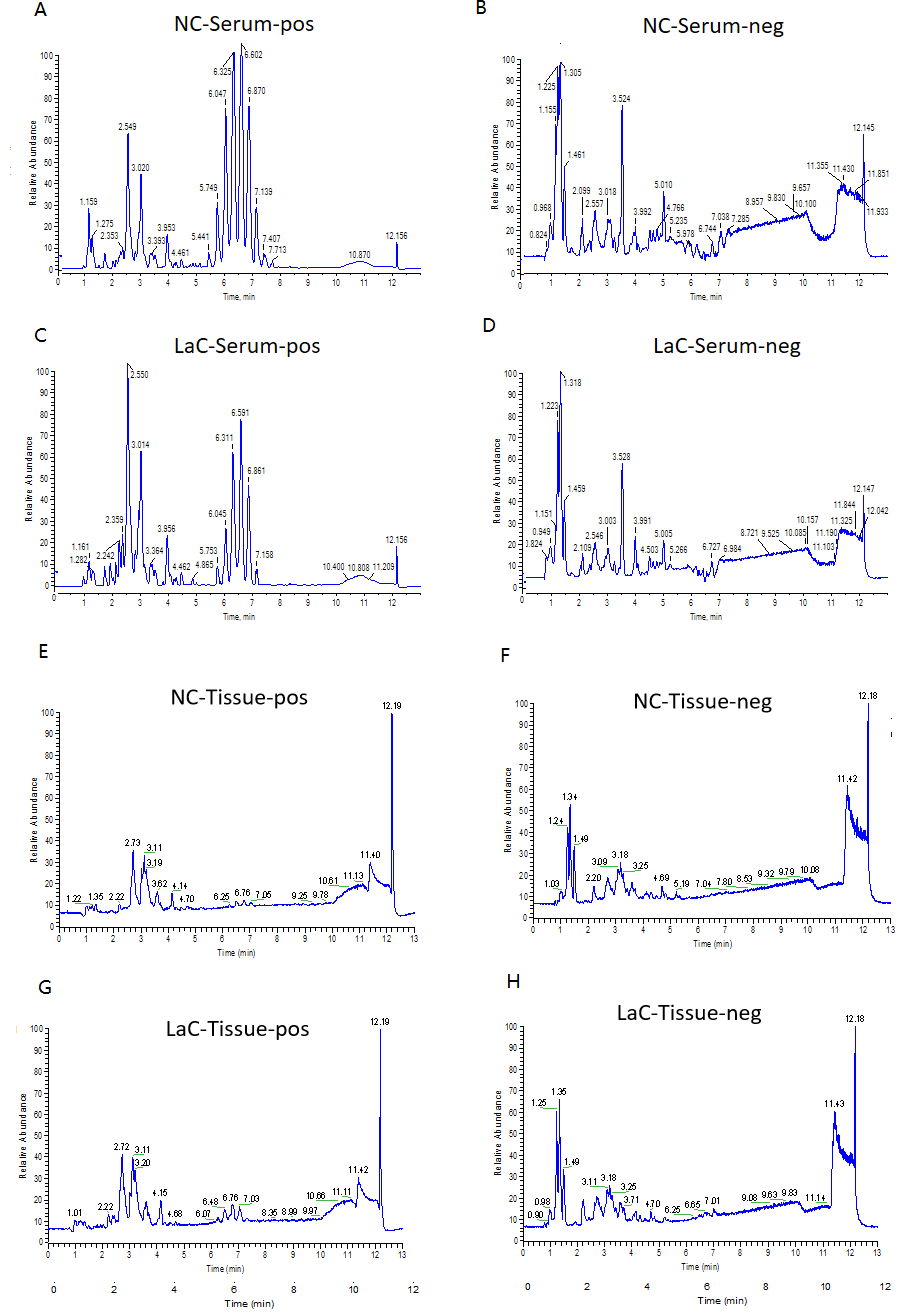


Figure S1. The total ion current chromatograms of serum and tissue from LaC and NC subjects in both positive and negative ion modes, respectively.


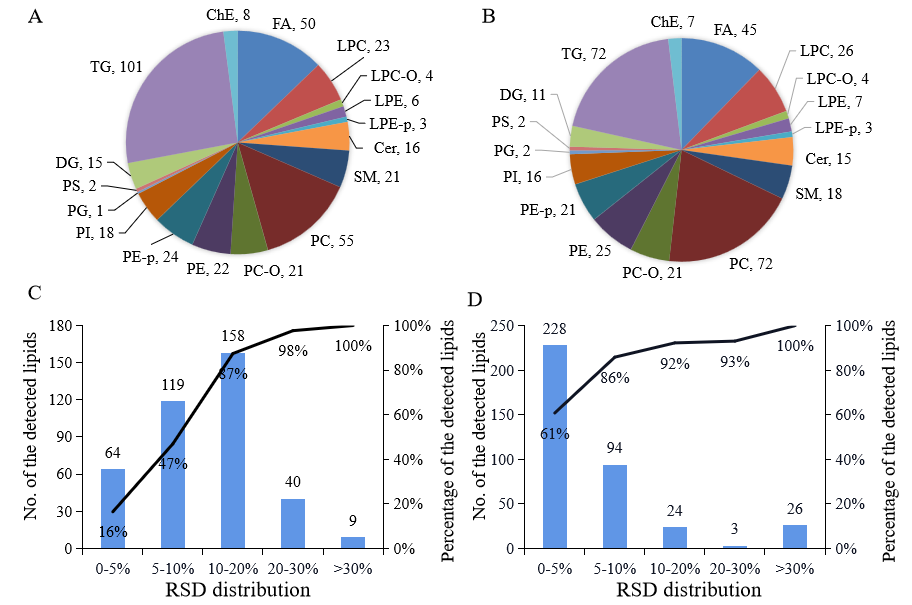


Figure S2. Distribution of all identified lipid species (subclass, no. of lipids) in serum (A) and tissue (B) samples, respectively. RSD distribution of the identified lipids in all QC samples from serum (C) and tissue (D) samples, respectively.

Table S1. Information on lipid internal standards that were spiked in sample matrix prior to lipidom extraction

| **Lipid internal standards** | **Corrected lipid (sub)classes** | **Work solution (µg/mL)** | **Stock solution (mg/mL)** | **ESI (+) M/Z** | **ESI (-) M/Z** |
| --- | --- | --- | --- | --- | --- |
| FFA (16-d3) | FA, OAHFA | 0.7 | 1 | / | 258.2518 |
| LPC (19:0) | LPC, LPE, LPC-O, LPE-p | 0.35 | 1.25 | 538.3859 | 596.3925 |
| Cer (d18:1/12:0) | Cer, CerG1, CerG2 | 0.2 | 1 | 552.5346 | 550.5219 |
| SM (d18:0/17:0) | SM | 0.2 | 1 | 647.5118 | 705.5191 |
| PC (19:0/19:0) | PC, PC-O | 0.7 | 1 | 818.6650 | 876.6716 |
| PE (17:0/17:0) | PE, PE-p, PI, PS, PG | 0.35 | 1 | 720.5519 | 718.5373 |
| TG (15:0/15:0/15:0) | TG, DG, ChE | 0.5 | 0.5 | 782.7220 | / |

Table S2. The list of exact m/z values and retention times of all the lipid species identified.

Detiled information was shown in the seperated excel

Table S3. The details of the 204 differential lipids contributed to distigushing the LaC group from the NC group

| **Sample ID** | **LaC *vs.* NC** | | |
| --- | --- | --- | --- |
|  | **P value** | **FDR** | **FC** |
| Cer(d18:1/16:0) | 3.7E-04 | 9.5E-04 | 0.79 |
| Cer(d18:1/24:0) | 5.6E-03 | 1.1E-02 | 1.22 |
| Cer(d18:1/24:1) | 7.1E-03 | 1.4E-02 | 1.20 |
| Cer(d18:2/22:0); Cer(d16:1/24:1) | 1.9E-03 | 4.3E-03 | 1.29 |
| Cer(d18:2/24:1) | 1.7E-03 | 3.8E-03 | 1.37 |
| CerG1(d18:1/16:0) | 5.6E-03 | 1.1E-02 | 1.20 |
| CerG1(d18:1/22:0) | 2.0E-03 | 4.4E-03 | 1.28 |
| CerG1(d18:1/24:0) | 6.7E-05 | 2.1E-04 | 1.37 |
| CerG2(d18:2/16:0) | 9.7E-08 | 6.3E-07 | 1.85 |
| ChE 18:1 | 3.7E-04 | 9.5E-04 | 1.34 |
| ChE 18:3 | 4.9E-04 | 1.3E-03 | 1.59 |
| ChE 20:2 | 3.5E-03 | 7.5E-03 | 1.33 |
| ChE 20:3 | 1.1E-03 | 2.5E-03 | 1.41 |
| ChE 20:4 | 8.0E-03 | 1.6E-02 | 1.27 |
| DG 38:7 | 5.4E-03 | 1.1E-02 | 0.55 |
| DG(16:0_18:1) | 3.5E-11 | 5.8E-10 | 0.29 |
| DG(16:1_18:1); DG(16:0_18:2) | 1.2E-11 | 2.5E-10 | 0.32 |
| DG(16:1_18:2); DG(16:0_18:3) | 5.9E-09 | 5.2E-08 | 0.42 |
| DG(18:1_18:1) | 7.0E-07 | 3.6E-06 | 0.52 |
| DG(18:1_18:2) | 6.8E-08 | 4.4E-07 | 0.53 |
| DG(18:1_20:3) | 8.7E-06 | 3.3E-05 | 0.54 |
| DG(18:1_20:4) | 1.1E-04 | 3.3E-04 | 0.60 |
| DG(18:1_22:5); DG(18:2_22:4) | 3.0E-05 | 1.0E-04 | 0.53 |
| DG(18:1_22:6); DG(18:2_22:5) | 1.3E-03 | 3.1E-03 | 0.51 |
| DG(18:2_18:2); DG(18:1_18:3) | 1.9E-05 | 6.8E-05 | 0.52 |
| DG(18:2_20:3) | 1.3E-03 | 3.0E-03 | 0.56 |
| DG(18:2_20:4) | 6.9E-04 | 1.7E-03 | 0.64 |
| DG(18:2_22:6) | 7.2E-06 | 2.9E-05 | 0.42 |
| FA(12:0) | 2.2E-06 | 9.9E-06 | 0.70 |
| FA(14:0) | 6.3E-03 | 1.2E-02 | 0.76 |
| FA(14:1) | 1.3E-04 | 4.2E-04 | 0.60 |
| FA(15:0) | 3.3E-03 | 6.4E-03 | 0.75 |
| FA(16:0) | 9.3E-03 | 1.7E-02 | 0.86 |
| FA(18:4) | 9.7E-03 | 1.7E-02 | 0.68 |
| FA(20:0) | 1.8E-03 | 3.9E-03 | 0.82 |
| FA(20:4) | 2.6E-08 | 2.5E-07 | 0.49 |
| FA(20:5) | 6.4E-06 | 2.6E-05 | 0.41 |
| FA(20:6) | 1.9E-08 | 2.0E-07 | 0.79 |
| FA(22:0) | 3.9E-03 | 7.4E-03 | 0.79 |
| FA(24:4) | 2.4E-04 | 6.8E-04 | 0.72 |
| FA(24:5) | 1.6E-03 | 3.7E-03 | 0.75 |
| LPC(14:0) | 1.3E-06 | 6.0E-06 | 0.48 |
| LPC(15:0) | 5.0E-09 | 4.7E-08 | 0.45 |
| LPC(16:0) | 8.1E-12 | 2.1E-10 | 0.37 |
| LPC(16:1) | 4.3E-08 | 3.0E-07 | 0.62 |
| LPC(17:0) | 1.1E-11 | 2.5E-10 | 0.33 |
| LPC(18:0) | 7.4E-12 | 2.0E-10 | 0.32 |
| LPC(18:1) | 2.4E-09 | 2.5E-08 | 0.59 |
| LPC(18:3) | 6.8E-12 | 2.0E-10 | 0.42 |
| LPC(18:4) | 7.2E-06 | 2.9E-05 | 0.61 |
| LPC(20:0) | 5.4E-09 | 5.0E-08 | 0.46 |
| LPC(20:1) | 6.2E-12 | 2.0E-10 | 0.34 |
| LPC(20:2) | 1.9E-11 | 3.7E-10 | 0.41 |
| LPC(20:3) | 6.8E-12 | 2.0E-10 | 0.33 |
| LPC(20:4) | 1.6E-06 | 7.1E-06 | 0.60 |
| LPC(20:5) | 2.7E-05 | 1.1E-04 | 0.51 |
| LPC(22:0) | 6.6E-04 | 1.6E-03 | 0.56 |
| LPC(22:1) | 1.3E-05 | 4.7E-05 | 0.56 |
| LPC(22:3) | 9.8E-07 | 4.8E-06 | 0.50 |
| LPC(22:4) | 6.8E-12 | 2.0E-10 | 0.33 |
| LPC(22:6) | 2.2E-08 | 1.7E-07 | 0.48 |
| LPC(24:0) | 9.3E-03 | 1.8E-02 | 1.27 |
| LPC-O 16:1 | 6.8E-12 | 2.0E-10 | 0.41 |
| LPC-O 18:0 | 5.6E-12 | 2.0E-10 | 0.23 |
| LPC-O 18:1 | 5.6E-12 | 2.0E-10 | 0.20 |
| LPC-O 18:3 | 5.6E-12 | 2.0E-10 | 0.24 |
| LPE(16:0) | 2.8E-06 | 1.2E-05 | 0.70 |
| LPE(16:0p) | 5.6E-12 | 2.0E-10 | 0.23 |
| LPE(18:0) | 7.7E-11 | 1.1E-09 | 0.42 |
| LPE(18:0p) | 5.6E-12 | 2.0E-10 | 0.26 |
| LPE(18:1) | 3.3E-03 | 7.0E-03 | 0.77 |
| LPE(18:1p) | 4.7E-10 | 6.2E-09 | 0.30 |
| LPE(20:4) | 4.7E-08 | 3.2E-07 | 1.68 |
| OAHFA(18:2_22:6) | 1.5E-04 | 4.8E-04 | 0.61 |
| PC 34:0 | 2.4E-03 | 5.0E-03 | 0.78 |
| PC 36:6 | 3.2E-11 | 5.6E-10 | 2.68 |
| PC 36:7 | 3.4E-09 | 3.3E-08 | 2.91 |
| PC 38:7 | 3.6E-06 | 1.5E-05 | 1.35 |
| PC 38:8 | 4.1E-05 | 1.3E-04 | 1.83 |
| PC 39:3 | 1.2E-04 | 3.6E-04 | 1.60 |
| PC 40:8 | 1.8E-04 | 5.0E-04 | 1.76 |
| PC 40:9 | 3.3E-04 | 8.7E-04 | 1.53 |
| PC 42:4 | 2.4E-04 | 6.8E-04 | 1.77 |
| PC 42:5 | 1.9E-04 | 5.6E-04 | 1.47 |
| PC 42:6 | 5.1E-03 | 1.1E-02 | 1.67 |
| PC 44:10 | 3.3E-03 | 6.4E-03 | 1.42 |
| PC 44:4 | 9.8E-07 | 4.9E-06 | 2.18 |
| PC(14:0_18:2) | 2.0E-10 | 2.8E-09 | 3.30 |
| PC(15:0_18:1) | 1.6E-09 | 1.8E-08 | 2.94 |
| PC(15:0_18:2) | 9.2E-10 | 1.2E-08 | 2.42 |
| PC(15:0_20:4) | 6.3E-08 | 4.2E-07 | 2.12 |
| PC(15:0_22:6) | 1.9E-05 | 6.8E-05 | 1.72 |
| PC(16:0_14:0) | 6.4E-09 | 5.3E-08 | 2.69 |
| PC(16:0_15:0) | 2.0E-07 | 1.3E-06 | 2.21 |
| PC(16:0_16:0) | 1.2E-05 | 4.5E-05 | 1.45 |
| PC(16:0_16:1) | 2.2E-11 | 4.2E-10 | 4.23 |
| PC(16:0_18:1) | 1.1E-09 | 1.3E-08 | 2.31 |
| PC(16:0_18:2) | 5.4E-11 | 8.6E-10 | 1.82 |
| PC(16:0_20:3) | 6.4E-09 | 5.3E-08 | 1.93 |
| PC(16:0_20:4) | 2.4E-08 | 1.8E-07 | 1.93 |
| PC(16:0_20:5) | 1.1E-04 | 3.3E-04 | 1.79 |
| PC(16:0_22:4) | 5.3E-07 | 2.9E-06 | 1.87 |
| PC(16:0_22:5) | 5.0E-07 | 2.8E-06 | 1.79 |
| PC(16:1_18:2) | 6.5E-11 | 9.8E-10 | 2.46 |
| PC(16:1_18:3) | 1.2E-09 | 1.4E-08 | 3.76 |
| PC(16:2_18:2) | 1.5E-09 | 1.7E-08 | 4.95 |
| PC(17:0_18:1) | 8.5E-07 | 4.3E-06 | 2.00 |
| PC(17:0_20:4) | 2.8E-06 | 1.2E-05 | 1.79 |
| PC(17:1_18:1) | 3.7E-08 | 2.7E-07 | 1.91 |
| PC(18:0_18:1) | 6.1E-07 | 3.2E-06 | 1.89 |
| PC(18:0_18:2) | 8.7E-09 | 7.1E-08 | 1.82 |
| PC(18:0_20:2) | 1.0E-04 | 3.2E-04 | 1.50 |
| PC(18:0_22:4) | 1.5E-06 | 6.8E-06 | 1.64 |
| PC(18:0_22:5) | 8.4E-05 | 2.6E-04 | 1.80 |
| PC(18:1_20:4) | 1.7E-04 | 4.8E-04 | 1.48 |
| PC(18:1_22:6) | 5.2E-04 | 1.3E-03 | 1.37 |
| PC(18:2_18:2) | 3.4E-05 | 1.2E-04 | 1.73 |
| PC(18:2_20:1) | 1.3E-04 | 3.8E-04 | 1.63 |
| PC(18:2_20:4) | 2.7E-04 | 7.4E-04 | 2.67 |
| PC(19:0_18:2); PC(17:0_20:2) | 2.5E-07 | 1.5E-06 | 2.25 |
| PC(19:0_20:4) | 8.0E-07 | 4.1E-06 | 1.85 |
| PC(20:0_20:4) | 2.8E-04 | 7.7E-04 | 1.79 |
| PC(20:1_20:4) | 6.7E-05 | 2.1E-04 | 1.61 |
| PC(20:2_22:6) | 8.4E-04 | 2.1E-03 | 1.38 |
| PC(20:4_22:6) | 8.9E-05 | 2.7E-04 | 1.71 |
| PC-O 32:0 | 3.8E-07 | 2.2E-06 | 1.58 |
| PC-O 32:1 | 3.2E-08 | 2.4E-07 | 1.83 |
| PC-O 32:2 | 3.8E-07 | 2.2E-06 | 2.25 |
| PC-O 34:1 (FA18:1) | 2.2E-08 | 1.7E-07 | 1.72 |
| PC-O 34:2 (FA18:2) | 3.0E-05 | 1.0E-04 | 1.52 |
| PC-O 34:3 (FA18:2) | 1.0E-06 | 5.0E-06 | 1.68 |
| PC-O 35:3 | 9.3E-04 | 2.3E-03 | 1.70 |
| PC-O 36:2 (FA18:2) | 1.7E-03 | 3.8E-03 | 1.29 |
| PC-O 36:3 (FA18:2) | 3.3E-04 | 8.7E-04 | 1.41 |
| PC-O 36:4 (FA20:3) | 8.7E-06 | 3.3E-05 | 1.51 |
| PC-O 36:5 (FA20:4) | 4.6E-07 | 2.6E-06 | 1.73 |
| PC-O 38:3 (FA20:3) | 3.0E-04 | 8.0E-04 | 1.40 |
| PC-O 38:4 (FA20:4) | 1.1E-03 | 2.5E-03 | 1.36 |
| PC-O 38:5 (FA20:4) | 8.7E-06 | 3.3E-05 | 1.47 |
| PC-O 38:6 (FA22:5) | 3.2E-06 | 1.4E-05 | 1.68 |
| PC-O 38:7 (FA22:6) | 1.1E-03 | 2.5E-03 | 1.36 |
| PC-O 40:4 (FA20:4) | 4.8E-05 | 1.6E-04 | 1.48 |
| PC-O 40:5 (FA22:5) | 6.4E-05 | 2.0E-04 | 1.51 |
| PC-O 40:7 (FA22:6) | 1.2E-03 | 2.7E-03 | 1.39 |
| PC-O 40:8 (FA22:6) | 5.4E-05 | 1.7E-04 | 1.32 |
| PE(16:0_16:1) | 2.3E-09 | 2.9E-08 | 3.85 |
| PE(16:0_18:1) | 3.0E-07 | 1.8E-06 | 2.09 |
| PE(16:0_18:2) | 2.6E-06 | 1.2E-05 | 1.81 |
| PE(16:0_18:3) | 4.3E-05 | 1.4E-04 | 2.02 |
| PE(16:0_20:4) | 1.0E-06 | 5.0E-06 | 1.83 |
| PE(16:0_20:5) | 2.6E-03 | 5.7E-03 | 1.77 |
| PE(16:0_22:5) | 3.8E-05 | 1.4E-04 | 1.81 |
| PE(16:0_22:6) | 9.3E-03 | 1.8E-02 | 1.34 |
| PE(16:0p_18:1) | 3.0E-03 | 5.9E-03 | 1.38 |
| PE(16:0p_18:2) | 8.9E-05 | 3.1E-04 | 1.82 |
| PE(16:0p_20:4) | 1.9E-04 | 5.6E-04 | 1.69 |
| PE(16:0p_22:4) | 3.5E-03 | 7.5E-03 | 1.49 |
| PE(16:0p_22:5) | 1.4E-04 | 4.6E-04 | 1.68 |
| PE(17:0_18:2) | 3.0E-03 | 5.9E-03 | 1.54 |
| PE(18:0_18:2) | 7.4E-03 | 1.5E-02 | 1.41 |
| PE(18:0_20:3) | 1.7E-04 | 4.8E-04 | 1.68 |
| PE(18:0_22:5); PE(18:1_22:4) | 1.8E-03 | 4.0E-03 | 1.57 |
| PE(18:0p_20:4) | 3.5E-03 | 6.9E-03 | 1.48 |
| PE(18:0p_22:5); PE(20:0p_20:5) | 8.0E-04 | 1.9E-03 | 1.65 |
| PE(18:1_20:3) | 2.3E-05 | 8.1E-05 | 1.63 |
| PE(18:1_20:4) | 6.3E-04 | 1.6E-03 | 1.62 |
| PE(18:1p_18:2) | 8.0E-03 | 1.4E-02 | 1.53 |
| PE(18:1p_20:4) | 1.6E-03 | 3.7E-03 | 1.49 |
| PE(18:2_18:2) | 4.0E-03 | 8.4E-03 | 0.61 |
| PE(18:2_20:4) | 1.4E-04 | 4.2E-04 | 1.83 |
| PE(20:0p_18:2) | 8.3E-03 | 1.6E-02 | 1.53 |
| PE(20:0p_20:4) | 6.3E-04 | 1.6E-03 | 1.67 |
| PG(18:0_18:2) | 1.9E-08 | 2.0E-07 | 2.80 |
| PI(16:0_18:1) | 1.5E-03 | 3.4E-03 | 1.42 |
| PI(16:0_20:3); PI(18:1_18:2) | 1.8E-03 | 4.0E-03 | 1.39 |
| PI(16:0_20:4) | 1.5E-04 | 4.8E-04 | 1.60 |
| PI(17:0_18:1) | 7.5E-07 | 3.9E-06 | 2.48 |
| PI(17:0_18:2) | 3.7E-03 | 7.1E-03 | 1.32 |
| PI(17:0_20:4) | 4.5E-04 | 1.1E-03 | 1.57 |
| PI(18:0_18:2) | 2.6E-08 | 2.5E-07 | 2.57 |
| PI(18:0_20:3) | 1.4E-08 | 1.7E-07 | 2.68 |
| PI(18:0_22:4) | 2.7E-04 | 7.3E-04 | 1.39 |
| PI(18:1_18:1) | 7.1E-03 | 1.3E-02 | 2.16 |
| PI(18:1_20:4) | 3.1E-04 | 8.4E-04 | 1.50 |
| PI(19:0_20:4) | 3.4E-05 | 1.3E-04 | 1.51 |
| PS(21:0_18:2); PS(17:1_22:1) | 9.1E-08 | 6.7E-07 | 1.46 |
| SM 32:0 | 2.0E-03 | 4.4E-03 | 1.44 |
| SM 32:2 | 2.4E-04 | 6.7E-04 | 1.35 |
| SM 34:0 | 6.4E-06 | 2.6E-05 | 1.49 |
| SM 36:3 | 1.0E-03 | 2.5E-03 | 1.26 |
| SM 38:5 | 2.4E-04 | 6.7E-04 | 2.05 |
| SM 40:1 | 2.0E-03 | 4.3E-03 | 1.27 |
| SM 40:2 | 2.0E-03 | 4.4E-03 | 1.21 |
| SM 42:4 | 3.8E-05 | 1.3E-04 | 1.41 |
| SM(d16:1/16:0) | 5.7E-07 | 3.1E-06 | 2.01 |
| SM(d18:1/16:0) | 5.6E-06 | 2.3E-05 | 1.34 |
| SM(d18:1/18:0) | 1.0E-03 | 2.5E-03 | 1.31 |
| SM(d18:1/20:0) | 2.9E-05 | 1.0E-04 | 1.38 |
| SM(d18:1/24:0) | 4.0E-03 | 8.4E-03 | 1.23 |
| SM(d18:2/16:0) | 2.1E-09 | 2.2E-08 | 1.93 |
| SM(d18:2/18:0) | 1.5E-03 | 3.5E-03 | 1.25 |
| SM(d18:2/20:0) | 2.2E-06 | 9.7E-06 | 1.43 |
| SM(d18:2/24:1) | 1.7E-05 | 6.2E-05 | 1.35 |
| TG(16:1_18:2_20:5) | 8.0E-03 | 1.6E-02 | 1.94 |
